# Mechanical and aesthetics compatibility of Brazilian red propolis micellar nanocomposite as a cavity cleaning agent

**DOI:** 10.1186/s12906-018-2281-y

**Published:** 2018-07-18

**Authors:** Isabel Cristina Celerino de Moraes Porto, Dayse Chaves Cardoso de Almeida, Gabriela Vasconcelos Calheiros de Oliveira Costa, Tayná Stéphanie Sampaio Donato, Letícia Moreira Nunes, Ticiano Gomes do Nascimento, José Marcos dos Santos Oliveira, Carolina Batista da Silva, Natanael Barbosa dos Santos, Maria Luísa de Alencar e Silva Leite, Irinaldo Diniz Basílio-Júnior, Camila Braga Dornelas, Pierre Barnabé Escodro, Eduardo Jorge da Silva Fonseca, Regianne Umeko Kamiya

**Affiliations:** 1 Postgraduate Program in Health Research, Cesmac University Center, Rua Cônego Machado, 825, Farol, Maceió, Alagoas Brazil; 2 Department of Restorative Dentistry, Faculty of Dentistry, Federal University of Alagoas, Campus AC Simões, Av. Lourival Melo Mota, S/N, Tabuleiro do Martins, Maceió, Alagoas Brazil; 3 Laboratory of Quality Control of Drugs and Medicines, Postgraduate Program in Pharmaceutical Sciences, School of Nursing and Pharmacy, Federal University of Alagoas, Campus A. C, Simões, Maceió, Alagoas Brazil; 4 Laboratory of Applied Electrochemistry, Institute of Chemistry and Biotechnology, Federal University of Alagoas, Campus A. C, Simões, Maceió, Alagoas Brazil; 5 Department of Cariology, Faculty of Dentistry, Federal University of Alagoas, Campus AC Simões, Av. Lourival Melo Mota, S/N, Tabuleiro do Martins, Maceió, Alagoas Brazil; 6 Department of Dental Materials and Prosthodontics, Faculty of Dentistry, Universidade Estadual Paulista Júlio de Mesquita Filho-UNESP, Araraquara, São Paulo, Brazil; 7 Faculty of Veterinary Medicine, Federal University of Alagoas, Campus Arapiraca, Unit of Viçosa, Viçosa, Alagoas Brazil; 8 Laboratory of Bacteriology. Institute of Biological and Health Sciences, Federal University of Alagoas, Campus A. C, Simões, Maceió, Alagoas Brazil

**Keywords:** Red propolis, Dental caries, Cavity disinfectant, Micellar nanocomposites, Dental fillings, Antibacterial activity, Isoflavonoids, UPLC-DAD assay

## Abstract

**Background:**

Propolis is a natural substance produced by bees and is known to have antimicrobial activity. Our aim was to evaluate the antimicrobial effect of micellar nanocomposites loaded with an ethyl acetate extract of Brazilian red propolis as a cavity cleaning agent and its influence on the color and microtensile bond strength (μTBS) of the dentin/resin interface.

**Methods:**

An ultra-performance liquid chromatography coupled with a diode array detector (UPLC-DAD) assay was used to determine the flavonoids and isoflavones present in an ethyl acetate extract of Brazilian red propolis (EARP) and micellar nanocomposites loaded with EARP (MNRP). The antimicrobial activity of EARP and MNRP was tested against *Streptococcus mutans*, *Lactobacillus acidophilus*, and *Candida albicans*. One of the following experimental treatments was applied to etched dentin (phosphoric acid, 15 s): 5 μL of MNRP (RP3, 0.3%; RP6, 0.6%; or RP1, 1.0% *w*/*v*), placebo, and 2% chlorhexidine digluconate. Single Bond adhesive (3 M/ESPE) was applied and a 4-mm-thick resin crown (Z350XT, 3 M/ESPE) was built up. After 24 h, the teeth were sectioned into sticks for the μTBS test and scanning electron microscopy. Spectrophotometry according to the CIE L*a*b* chromatic space was used to evaluate the color. Data were analyzed using one-way ANOVA and the Tukey test or Kruskal-Wallis test and the same test for pairwise comparisons between the means (*P* < 0.05).

**Results:**

The UPLC-DAD assay identified the flavonoids liquiritigenin, pinobanksin, pinocembrin, and isoliquiritigenin and the isoflavonoids daidzein, formononetin, and biochanin A in the EARP and micellar nanocomposites. EARP and MNRP presented antimicrobial activity against the cariogenic bacteria *Streptococcus mutans* and *Lactobacillus acidophilus*, and for *Candida albicans*. Δ*E* values varied from 2.31 to 3.67 (*P* = 0.457). The mean μTBS for RP1 was significantly lower than for the other groups (*P* < 0.001). Dentin treated with RP1 showed the shortest resin tags followed by RP6 and RP3.

**Conclusions:**

The EARP and (MNRP) showed antimicrobial activity for the main agents causing dental caries (*Streptococcus mutans* and *Lactobacillus acidophilus*) and for *Candida albicans*. MNRP at concentrations of 0.3 and 0.6% used as a cavity cleaner do not compromise the aesthetics or μTBS of the dentin/resin interface.

## Background

Propolis is produced by bees to close small gaps in the hive, prevent entry of insects, and reduce the proliferation of fungi and bacteria. Therefore, it is an important natural antibiotic and plays a promising role in medicine and dentistry [1, 2].

Useful knowledge about propolis has been gathered from research in the fields of medicine, pharmacology, food sciences and chemistry [2, 3]. In dentistry, some work has been published on endodontics [4, 5], preventive dentistry [6, 7], cariology [3, 6], surgery [8], and periodontics [9, 10], but there is a shortage of studies in the field of restorative dentistry.

In Brazil, the biodiversity of the flora favors the emergence of 13 types of propolis. Red propolis (RP), the 13th type of Brazilian propolis with a characteristic intense red color, is produced by bees of the species *Apis mellifera* with the sap of *Dalbergia ecastophyllum*, a leguminous plant that inhabits the northeastern mangroves of Brazil [11]. Brazilian RP has unique components that differentiate it from other propolis produced in Brazil and around the world. Isoflavonoids, propolones/guttiferones, terpenes, chalcones, and phenolic compounds are the principal classes of secondary metabolites present in RP [2, 12, 13].

Alencar et al. [14] reported the presence of at least four isoflavones in Brazilian RP that have never been found before: homopterocarpin, medicarpin, 4′,7-dimethoxy-2′-isoflavonol, and 7,4′-dihydroxyisoflavone. Three other new compounds were identified by Awale et al. [15]: (6a*S*,11a*S*)-6a-ethoxymedicarpan, 2-(2′,4′-dihydroxyphenyl)-3-methyl-6-methoxybenzofuran, and 2,6-dihydroxy-2-[(4-hydroxyphenyl)methyl]-3-benzofuranone. In Brazilian RP from Alagoas state, a newer study [16] identified other unique components, such as 3,4,2′,3′-tetrahydroxychalcone and a flavone C-glycoside, not found before in propolis from other sources.

Brazilian RP has potent antimicrobial activity even at low concentrations (0.1 and 1.0%). Several studies have proved the effective action of Brazilian RP against *Streptococcus mutans* and *Lactobacillus* [3, 7, 17–19] attributed to the high concentration of flavonoids and phenolic compounds in Brazilian RP [20]. The best results were obtained with an acetone fraction of an alcoholic extract of Brazilian propolis, possibly because of its high polarity; the acetone fraction contains a greater amount of active phenolics, which increases the antimicrobial activity [21]. However, propolis is poorly soluble in water, and at higher concentrations (2, 10, 20, 30%) it causes unacceptable color changes [22] and obliteration of the dentinal tubules with possible damage to adhesive procedures [9].

A wide variety of new chemicals are emerging from natural products, and many active components extracted from them are water insoluble, which represents a great challenge for the development of new products. Micellar nanocomposites, which are submicrometer colloidal dispersions of pure drug particles that are stabilized by a small percentage of excipients, could dramatically enhance the solubility and dissolution rate of drug particles. Furthermore, micellar nanocomposites are the most suitable vehicle for drugs that require high doses or limited volume to be administered. Micellar nanocomposites are a promising technique for the development of poorly water-soluble drugs, especially for screening and early evaluation of candidate active pharmaceutical ingredients. In addition, they may provide many advantages for drug delivery, such as increased drug loading, improved drug absorption, and retention for various delivery routes [23].

Dental caries is considered a chronic and multifactorial disease and occurs as a result of the dissolution of tooth mineral by acids derived from bacterial fermentation of dietary carbohydrates, mainly by *Streptococcus mutans* and *Lactobacillus* ssp., which are involved in the initiation and progression of the lesions, respectively [24].

Contemporary dentistry seeks to arrest the evolution of caries and to prevent new lesions, with minimal restorative intervention and trying to preserve tooth vitality. Traditionally, caries treatment involves the removal of carious tissue and replacing it with a restorative material. However, conventional removal of carious tissue and cavity preparation procedures do not guarantee complete elimination of oral cariogenic bacteria that might be entrapped within the dentin tubules or the smear layer [25]. In addition, in deep cavities, the removal of large amounts of dentin can lead to pulp exposure, and consequently bacterial contamination, which may lead to the need for endodontic treatment [26]. Thus, the removal of only the infected carious dentin layer and preservation of the innermost layer, which can be re-mineralized, provides greater protection to the pulp and is therefore applicable when pulp exposure is imminent [26–29]. Greater benefits can be achieved through measures to eliminate the maximum amount of bacteria remaining in the dental tissue, such as disinfection of the cavity before the restorative procedure [30].

Because Brazilian RP contains waxes and resins and is an intense red color, its use as a natural antimicrobial agent in carious cavities is a challenge. Thus, it is important to investigate its influence on the bond strength and aesthetics of the restoration. The aim of this study was to evaluate the antimicrobial effect of micellar nanocomposites loaded with an ethyl acetate extract of Brazilian RP as a cavity disinfection agent and its influence on the color and microtensile bond strength of the dentin/resin interface.

## Methods

Fifty-four extracted, human, caries-free third molars were obtained from adult patients of both genders were stored in 0.5% chloramine T solution at 4 °C and used within 2 months of extraction. Written informed consent was obtained from all subjects. All procedures followed in this study were in accordance with the ethical principles of the Declaration of Helsinki.

### Preparation of Brazilian red propolis micellar nanocomposite

Brazilian RP raw material was collected from Marechal Deodoro, Alagoas, Brazil. Propolis was collected from the Ilha do Porto apiary (geographic coordinates 9° 44.555′ S, 35° 52.080′ W, and 18.1 m above sea level) during the month of July/2013. To obtain RP extract, raw propolis (250 g) was manually ground and placed in a flask with 600 mL of 80% ethanol, then placed on an agitator (Thornton, Model T14, Thornton Inpec Eletrônica Ltda., Vinhedo, Brazil) for 48 h. The macerate (the liquid portion) was removed using a pipette, and the solid portion (wax) was discarded. The macerate was mixed with 600 mL of 80% ethanol in a glass flask and agitated for 24 h. The resulting macerate was again mixed with 600 mL of 80% ethanol and left for 24 h without agitation. Next, the macerate was removed using a pipette, filtered through filter paper, and subjected to distillation under reduced pressure in a rotary evaporator (model 801/802; Fisatom, São Paulo, Brazil) in a water bath at 80–90 °C (pressure 650 mmHg and speed 80 rpm) to remove the solvent. The extract of Brazilian RP was then placed in a glass container and left for approximately 3 days for the residual solvent to evaporate; a viscous solid mass (162 g) was obtained as a crude extract of RP.

Liquid-liquid extraction of this crude extract was performed to eliminate grease and waxes. The crude extract (8 g) was solubilized with absolute ethanol (35 mL) and 15 mL of distilled water was added in a beaker. This RP crude extract was transferred to a separation funnel and hexane (50 mL) was added to eliminate the grease and wax present in the crude extract. The hexane layer was removed with a separation funnel and then ethyl acetate solvent (200 mL) was added in two liquid-liquid extraction steps to obtain an ethyl acetate extract enriched with the flavonoids and isoflavonoids from the RP, free of grease and wax. The ethyl acetate extract was subjected to distillation under reduced pressure in a rotary evaporator to obtain a solid mass (4.0 g), which was used in all the experiments in this study.

The experimental micellar nanocomposites were prepared as follows. One hundred milligrams of the polymeric nanocomposites of poly-ε-caprolactone (PCL, molecular weight 10,000) and Pluronic F108 copolymer (molecular weight 14,000) in a proportion of 7:3 were weighed and mixed with 100 mg (RP1) or 60 mg (RP6), or 30 mg (RP3) of ethyl acetate extract of Brazilian red propolis (EARP). Then, 8 mL of acetone was enough to solubilize the micellar nanocomposites loaded with EARP under agitation in an ultrasonic bath for 10 min. The final volume was adjusted to 10 mL with acetone to obtain 1.0, 0.6, and 0.3% nanocomposites in a micellar state (≈70 nm).

### Determination of Brazilian red propolis markers using the UPLC-DAD method

The identification and quantification of markers in the EARP and 1% micellar nanocomposites loaded with EARP were performed using ultra-performance liquid chromatography coupled with a diode array detector (UPLC-DAD) from Shimadzu (Tokyo, Japan). The equipment consisted of the following modules: a high-pressure pump (model LC-20ADXR), degasser (model DGU-20A3R), auto-injector (model SIL-20AXR), oven chromatographic column, photodiode array detector (model EPDM-20A), a controller (model CBM-20A), and Shimadzu Labsolution software.

The separation of flavonoids occurred using a reversed-phase column (C_18_, 150 mm 4.6 mm; 5 μm), and a mobile phase that consisted of solvent A (Milli-Q water) and solvent B (acetonitrile), pumped at a flow rate of 0.3 mL/min. The initial elution gradient consisted of 70% water (A) and 30% acetonitrile (B) (*v*/v). The column was eluted by varying the percentage of (B) as follows: 0–2 min 30% B, 2–5 min 36% B, 5–8 min 46% B, 8–11 min 52% B, 11–14 min 52% B, 14–17 min 57% B, 17–20 min 62% B, 20–24 min 62% B, 24–28 min 68% B, 28–32 min 72% B, 32–36 min 90% B, 36–42 min 97% B, 42–50 min 100% B, 50–55 min 100% B, 55–57 min acetonitrile was reduced to 30% and this condition was maintained up to 60 min. This method was based on Nascimento et al. [31]. This long method was developed in order to wash the column during the analysis with 100% acetonitrile, avoid lack of accuracy and loss of precision during the entrapment assay, and avoid column fouling and excessive pressure buildup by irreversible retention of non-polar compounds (terpenes and guttiferones present in Brazilian RP extract). The injection volume was 2 μL. Analytical standards of the flavonoids described as markers (daidzein, liquiritigenin, pinobanskin, isoliquiritigenin, formononetin, pinocembrin, and biochanin A), the EARP, and 1% micellar nanocomposites loaded with EARP were prepared in a stock solution of 10,000 μg/mL, using acetone as solvent and diluted to a concentration of 500 μg/mL. Calibration of the marker quantification method was carried out according to Nascimento et al. [31].

### Antimicrobial activity

The EARP, micellar nanocomposite loaded with EARP (MNRP), 0.12% chlorhexidine gluconate (Ao Pharmacêutico, Unidade Maceió, Brazil), and 0.2% triclosan (Ao Pharmacêutico, Unidade Maceió, Brazil) were tested against *Streptococcus mutans* CCT 3440, *Lactobacillus acidophilus* ATCC 4356, and *Candida albicans* ATCC 36801 and 36,802. The strains from Tropical Cultures Collection (CCT) and American Type Culture Collection (ATCC) were provided by Oswaldo Cruz Foundation, Rio de Janeiro, Brazil. All strains were activated in their selective medium, MSB agar (mitis salivarius-bacitracin, 0.2 U/L), Lactobacilli MRS Agar (deMan, Rogosa and Sharpe), and Candida BCG agar (bromocresol green containing cloramphenicol 0.5 g/L), respectively.

The minimal inhibitory concentration (MIC) test or broth microdilution assay was carried out using microplates (96 wells), following the procedure according to the Clinical and Laboratory Standards Institute [32] and Lima et al. [33] for bacteria and EUCAST [34] for yeast, with some modifications. The McFarland Scale 0.5 determined the cell concentration. A standardized inoculum containing about 10^6^ bacteria/mL or 10^5^ yeast/mL of a pure overnight culture was used in the tests. Serial two-fold dilutions (range from 15.62 μg/mL to 1000 μg/mL) of Ethyl acetate extract of Brazilian RP and MNRP were prepared in 80 μL of Brain Heart Infusion broth (BHI) or Mueller Hinton broth (MHB) in a 96-well microplate. Both ethyl acetate and micellar nanocomposite without Brazilian RP (blank) were also tested for each serial dilution. Microbial cultures were grown in medium without antimicrobial samples (negative control) and with 0.12% chlorhexidine gluconate or 0.2% triclosan (positive controls). Then, 20-μL aliquots of standardized inoculum were added to the wells to give a final volume of 100 μL/well and serial two-fold dilution (15.62, 31.25, 62.50, 125, 250, 500 and 1000 μg/mL). *Candida albicans* were incubated at 37 °C for 24 h in aerobic conditions. *Streptococcus mutans* and *Lactobacillus acidophilus* were incubated in a microaerobic environment at 37 °C for 24 h. The growth of bacterial cells was measured by absorbance at OD_550–630_ nm in an automated microplate reader (Bio-Rad 680, Madison, WI, USA).

The MIC values were defined as the lowest bandwidth concentration of the tested compounds that inhibited 100% of bacterial growth compared with the negative control. All samples were assayed in quadruplicate in three independent experiments. An aliquot (30 μL) of a concentration higher than MIC was cultured on respective selective media for 24 h, at 37 °C to determine the Minimal bactericidal concentration (MBC). MBC was the lowest concentration that allowed no visible bacterial growth on agar.

### Color analysis

The teeth were cut perpendicular to the long axis at the level of the enamel–dentin junction with a diamond disc (Extec, Enfield, CT, USA) fitted in a metallographic cutter (Extec Technologies Inc., Enfield, CT, USA) to obtain a flat dentin surface. The exposed dentin surface was wet-abraded with 600-grit silicon carbide paper for 60 s to create a standardized smear layer. Then, the dentin surfaces were examined under a stereomicroscope at 40× magnification for the presence of enamel.

The teeth were randomly divided into three groups (*n* = 10) according to the treatment: micellar nanocomposites loaded with EARP (RP3, 0.3%; RP6, 0.6%; or RP1, 1.0% *w*/*v*). The teeth were sectioned mesiodistally with the same diamond disc under water lubrication to obtain 60 half teeth and then attached to a wax flat base with a thin metal plate between each pair to ensure that only one half tooth half received the experimental treatment. The other half tooth was the negative control (no treatment, baseline).

In this stage of the study, we used two techniques, before and after acid etching of dentin, to apply micellar nanocomposites loaded with EARP: (1) before etching, using propolis micellar nanocomposites as a cavity cleaner; (2) after etching to improve the effect of propolis, which can be totally or partially removed during etching.

The micellar nanocomposites loaded with EARP were applied on the dentin surface for 1 min, which was then etched for 15 s with 37% phosphoric acid (Atack Tec, Caithec Indústria Ltd., Rio do Sul. SC, Brazil) or was applied on etched dentin before application of Adper Single Bond 2 adhesive (3 M do Brasil Ltd., Sumaré, SP, Brazil) followed by a covering of Filtek Z 350XT composite resin (3 M do Brasil Ltda, Sumaré, SP, Brazil). The adhesive and the composite resin were irradiated according to the manufacturer’s instructions with an LED device (1150 mW/cm^2^; Emitter B; Schuster Com. Equip. Odontológicos Ltda, Santa Maria, RS, Brazil) and stored in distilled water at 37 °C for 24 h.

Color was measured with a spectrophotometer (Minolta CR-321, Konika Minolta, Tokyo, Japan), ensuring that the readings were taken with the half tooth in the same position. The spectrophotometer converted the information obtained from the bonded surface to a digital scale in the CIELAB system (L* a* b*) [35, 36]. In order to limit the area for color readings to the bonded surface, the size of the reading window was reduced to a circumference of approximately 1 mm using black opaque tape. The placebo (micellar nanocomposite without Brazilian red propolis [DL]) and 2% chlorhexidine digluconate (CHX) were not tested because they are colorless.

### Microtensile bond strength test

The teeth were divided into groups according to the dentin pretreatment: micellar nanocomposites loaded with EARP 0.3% (RP3); micellar nanocomposites loaded with EARP 0.6% (RP6); micellar nanocomposites loaded with EARP 1.0% (RP1); DL; 2% CHX (FGM, Joinville, Brazil); and no treatment (NT; negative control group). The same restorative procedure was used throughout. The placebo micellar nanocomposite (DL) was used to separate the effect of the micellar nanocomposites loaded with EARP from the effect produced by the EARP.

After storing in distilled water at 37 °C for 24 h, the bonded specimens were serially sectioned using a low-speed diamond saw (Extec Technologies, Inc., Enfield, CT, USA) across the bonded interface in both the x and y directions into beams with a cross-sectional area of ~ 0.8 mm^2^. These specimens were individually attached to a custom-made testing jig with cyanoacrylate glue and were subjected to microtensile testing at a crosshead speed of 0.1 mm/min until failure using a Microtensile OM 100 tester (Odeme, Luzerna, SC, Brazil). Fractured specimens were examined with a stereomicroscope at 40× magnification (Coleman Co. Ltd., Santo André, SP, Brazil) to determine the mode of failure. Selected debonded sticks were observed by scanning electron microscopy (SEM).

### Scanning electron microscopy

Two specimens (sticks) from each group were prepared for examination by SEM in order to evaluate the morphology of the dentin/resin interface. The specimens were polished using ascending SiC papers from 600 up to 1200 grit. Further final polishing was performed using 1.00 μm and 0.05 μm alumina suspensions (Buehler, Lake Bluff, IL, USA). The specimens were cleaned in an ultrasonic bath containing distilled water for 15 min at each polishing step. Each specimen was fixed in Karnovsky solution for 12 h, washed in running water for 1 h, etched with 37% phosphoric acid for 1 min to facilitate the observation of the hybrid layer on the dentin/resin interface, then deproteinized with 10% sodium hypochlorite for 5 min, and rinsed with distilled water before being dehydrated in ascending concentrations of ethanol (25, 50, 75, 95, and 100%), followed by soaking in hexamethyldisilazane for 10 min and left to dry for 24 h at room temperature. The specimens were fixed in stubs using adhesive copper-conducting tape and examined at a variety of magnifications in a scanning electron microscope (TM-3000, Hitachi High Technologies, Tokyo, Japan).

### Statistical analysis

The color change data were analyzed using one-way factorial analysis of variance (ANOVA) followed by the Tukey test. Microtensile bond strength data were analyzed by Kruskal-Wallis test and the same test was used for pairwise comparisons between the means [37]. The Statistical Package for the Social Sciences, version 21 (SPSS, Chicago, IL, USA) and MEDCALC, version 12.5.0 (MedCalc Software, Acacialaan, Ostend, Belgium) were used for all statistical analyses (α = 0.05).

## Results

**Fig. 1:**
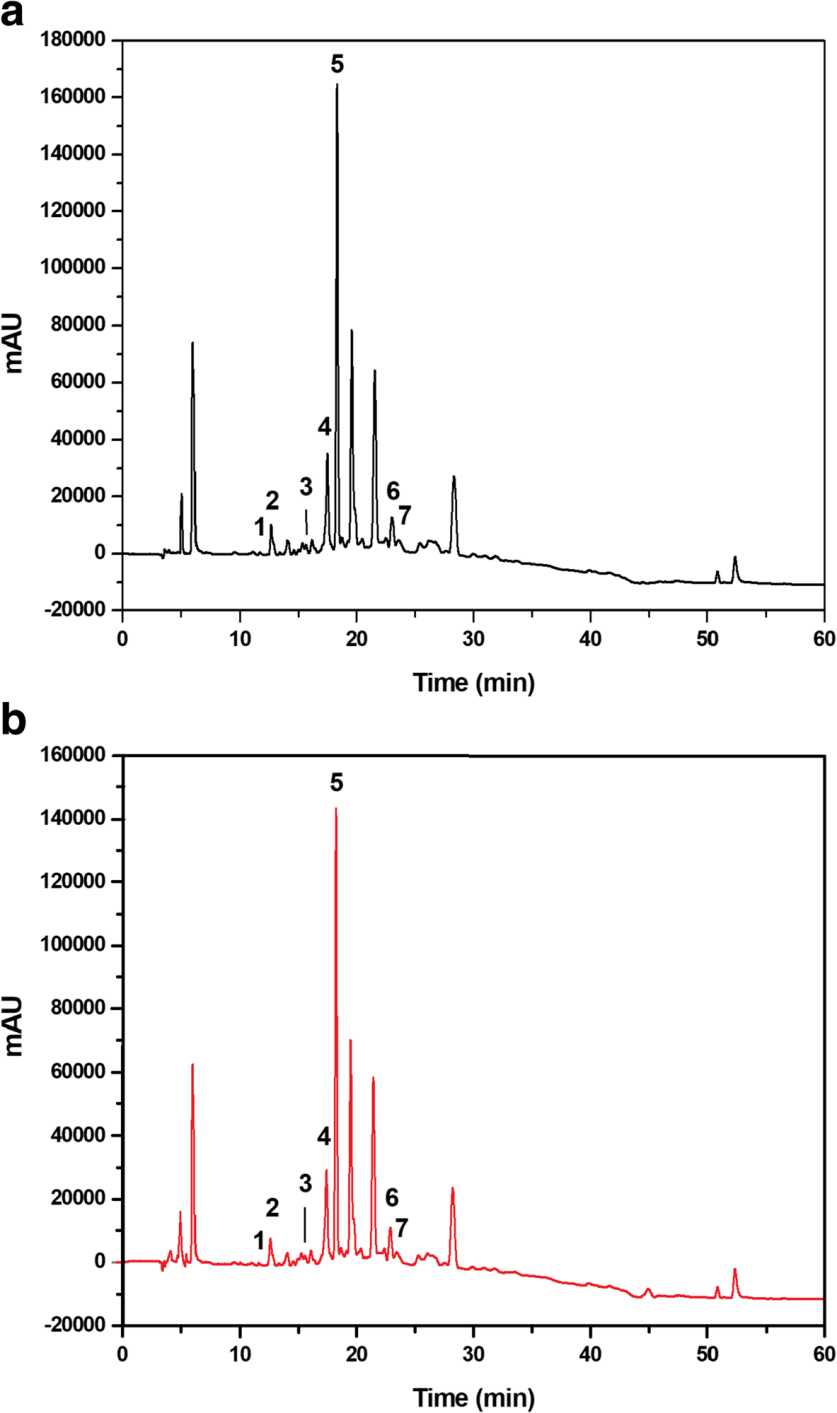
Markers of Brazilian red propolis extract using UPLC-DAD. Chromatograms of an ethyl acetate extract of Brazilian red propolis (**a**, black line) and 1% micellar nanocomposite loaded with EARP (**b**, red line) at a concentration of 500 μg/mL. Identification of daidzein (1), liquiritigenin (2), pinobanskin (3), isoliquiritigenin (4), formononetin (5), pinocembrin (6), and biochanin A (7) flavonoids in the UPLC-DAD profile at a wavelength of 205 nm

The overlapping chromatograms obtained using the UPLC-DAD technique for the EARP and micellar nanocomposites loaded with EARP are shown in Fig. 1. The corresponding retention times and peak purity at the maximum wavelengths were determined and compared with the analytical standards of flavonoids (Fig. 1a). The markers daidzein (1), liquiritigenin (2), pinobanksin (3), isoliquiritigenin (4), formononetin (5), pinocembrin (6), and biochanin A (7) were identified and quantified using the UPLC-DAD profile of the EARP and the 1% micellar nanocomposites loaded with EARP (Fig. 1b). The values for the corresponding retention times, maximum wavelengths and concentrations of the flavonoids markers in the EARP and 1% micellar nanocomposites loaded with EARP (RP1) are shown in Table 1.

**Table 1:** Chromatographic parameters: retention times (RT), maximum wavelength, and concentration of ethyl acetate extract of Brazilian red propolis and 1% of the micellar nanocomposite loaded with EARP using UPLC-DAD

Marker	RT (min)	λ (nm)	Concentration (μg/mL)	Concentration % (*w*/w)
Ethyl acetate extract of Brazilian red propolis: 500 μg/mL				
Daidzein	11.67	249	0.399	0.079
Liquiritigenin	12.68	275	7.131	1.426
Pinobanksin	15.78	289	0.385	0.077
Isoliquiritigenin	17.41	366	5.123	1.024
Formononetin	18.23	249	5.841	1.168
Pinocembrin	22.88	289	1.335	0.267
Biochanin A	23.81	260	0.315	0.063
1% micellar nanocomposite loaded with EARP: 500 μg/mL				
Daidzein	11.67	249	0.179	0.035
Liquiritigenin	12.68	275	6.082	1.216
Pinobanksin	15.78	289	0.299	0.059
Isoliquiritigenin	17.41	366	4.417	0.883
Formononetin	18.23	249	4.990	0.998
Pinocembrin	22.88	289	1.125	0.225
Biochanin A	23.81	260	0.281	0.056

*EARP* ethyl acetate extract of Brazilian red propolis

The results of the MIC determination of the EARP and micellar nanocomposite loaded with EARP are presented in Table 2. Both 0.12% chlorhexidine and 0.2% triclosan inhibited the growth of all microorganisms at the concentration range tested. The ethyl acetate solvent without propolis extract and the micellar nanocomposite without EARP (blank) did not inhibit the microorganisms. *Lactobacillus acidophilus* ATCC 4356, *Streptococcus mutans* CCT 3440, *Candida albicans* ATCC 36801, and *Candida albicans* 36,802 were sensitive to the EARP (32–125 μg/mL) and MNRP (15.62–1000 μg/mL) at concentrations below the lowest concentration used in this study (i.e., 3000 μg/mL). The MIC of MNRP against *Lactobacillus acidophilus* ATCC 4356 was found to be < 15.62 μg/mL.

**Table 2:** Minimal inhibitory concentration (MIC) and minimal bactericidal concentration (MBC) of ethyl acetate extract of Brazilian red propolis and micellar nanocomposite loaded with Brazilian red propolis for *Streptococcus mutans*, *Lactobacillus acidophilus*, and *Candida albicans*

Microorganisms	MIC (μg/mL)		MBC (μg/mL)	
	EARP	MN	EARP	MN
*Candida albicans* ATCC 36801	31.25–62.50	250–500	500–1000	> 1000
*Candida albicans* ATCC 36802	31.25–62.50	250–500	250–500	> 1000
*Streptococcus mutans* CCT 3440	31.25–62.50	250–500	125–250	> 1000
*Lactobacillus acidophilus* ATCC 4356	31.25–62.50	< 15.62	62.5–125	125–250

*EARP* ethyl acetate extract of Brazilian red propolis, *MN* micellar nanocomposites loaded with EARP. Mueller Hinton Broth (MHB) was used in the antimicrobial test of *Candida albicans* and Brain Heart Infusion Broth (BHI) was used for *S.mutans* and *L. acidophilus*

The mean Δ*E* values and standard deviations for all groups are shown in Table 3. The Δ*E* values ranged from 2.31 to 3.67 and showed no significant differences among the groups regarding the propolis protocol, before or after etching (*P* = 0.457). Among all the micellar nanocomposites tested, major color changes were observed in the groups treated with 0.6 and 1.0% micellar nanocomposites loaded with EARP applied after dentin etching.

**Table 3:** Mean values of total color variation (∆*E*) according to treatment and processing technique

Treatment	Color variation (∆*E*)			
	Micellar nanocomposites loaded with EARP applied before dentin etching		Micellar nanocomposites loaded with EARP applied after dentin etching	
	Mean	Standard deviation	Mean	Standard deviation
Micellar nanocomposites 0.3% (RP3)	2.58	0.99	2.31	1.10
Micellar nanocomposites 0.6% (RP6)	2.67	1.89	3.67	2.67
Micellar nanocomposites 1.0% (RP1)	2.31	1.01	3.30	1.62

*EARP* ethyl acetate extract of Brazilian red propolis

There was no significant difference in the total color change (Δ*E*) between the processing techniques (before or after etching) or among the micellar nanocomposites loaded with EARP (i.e., RP3, RP6, and RP1) on pairwise comparisons considering the same application technique.

The values of the CIE L*a* b*coordinates (Table 4), regardless of the processing technique, do not show significant differences in the L* coordinate between the RP6 and RP1 groups. There was no significant difference for the a* coordinate (before etching), however, comparing RP3 with RP6 and RP1 (after etching), the difference was statistically significant. For the b* coordinate, the RP6 group before etching and RP1 after etching showed significant differences from all other experimental groups.

**Table 4:** Means ± standard deviation of the color characteristics in the L*a*b* color space (CIELAB) for each experimental group and the control groups

Treatment	Micellar nanocomposites loaded with EARP applied before dentin etching		Micellar nanocomposites loaded with EARP applied after dentin etching			
	L*	a*	b*	L*	a*	b*
Micellar nanocomposites 0.3% (RP3)	45.46 ± 2.46^Aa^	− 1.08 ± 1.09^Aa^	3.83 ± 1.67^Aa^	47.21 ± 2.60^Aa^	− 2.05 ± 0.42^Ab^	3.66 ± 1.32^Aa^
RP3 control	45.02 ± 3.04^Aa^	−1.33 ± 0.53^Aa^	3.39 ± 1.89^Aa^	48.90 ± 1.94^Ab^	− 2.25 ± 0.44^Ab^	3.93 ± 1.23^Aa^
Micellar nanocomposites 0.6% (RP6)	43.50 ± 1.11^Ba^	− 0.93 ± 0.62^Aa^	0.98 ± 1.22^Ba^	43.28 ± 3.75^Ba^	− 0.89 ± 0.88^Ba^	2.76 ± 0.89^Aa^
RP6 control	44.85 ± 2.35^Ba^	− 1.61 ± 0.45^Aa^	1.84 ± 1.63^Aa^	46.20 ± 1.87^Aa^	− 1.17 ± 0.52^Aa^	2.84 ± 1.60^Aa^
Micellar nanocomposites 1.0% (RP1)	44.44 ± 2.41^Ba^	− 1.61 ± 0.53^Aa^	3.30 ± 1.38^Aa^	43.33 ± 2.31^Ba^	− 0.94 ± 0.32^Ba^	1.63 ± 1.74^Ba^
RP1 control	46.02 ± 2.58^Aa^	− 1.70 ± 0.65^Aa^	3.04 ± 1.02^Aa^	45.46 ± 2.92^Aa^	− 1.55 ± 0.48^Aa^	2.44 ± 1.54^Aa^

F test (ANOVA) followed by the Tukey test for paired comparisons of L*, a*, and b* CIELAB coordinates. When all the capital letters (columns) are different, there is a significant difference between color changes for each CIELAB coordinate. When all the lower case letters (rows) are different, there is a significant difference between different processing techniques in the same CIELAB coordinate by Tukey’s paired comparisons. EARP, ethyl acetate extract of Brazilian red propolis

**Fig. 2:**
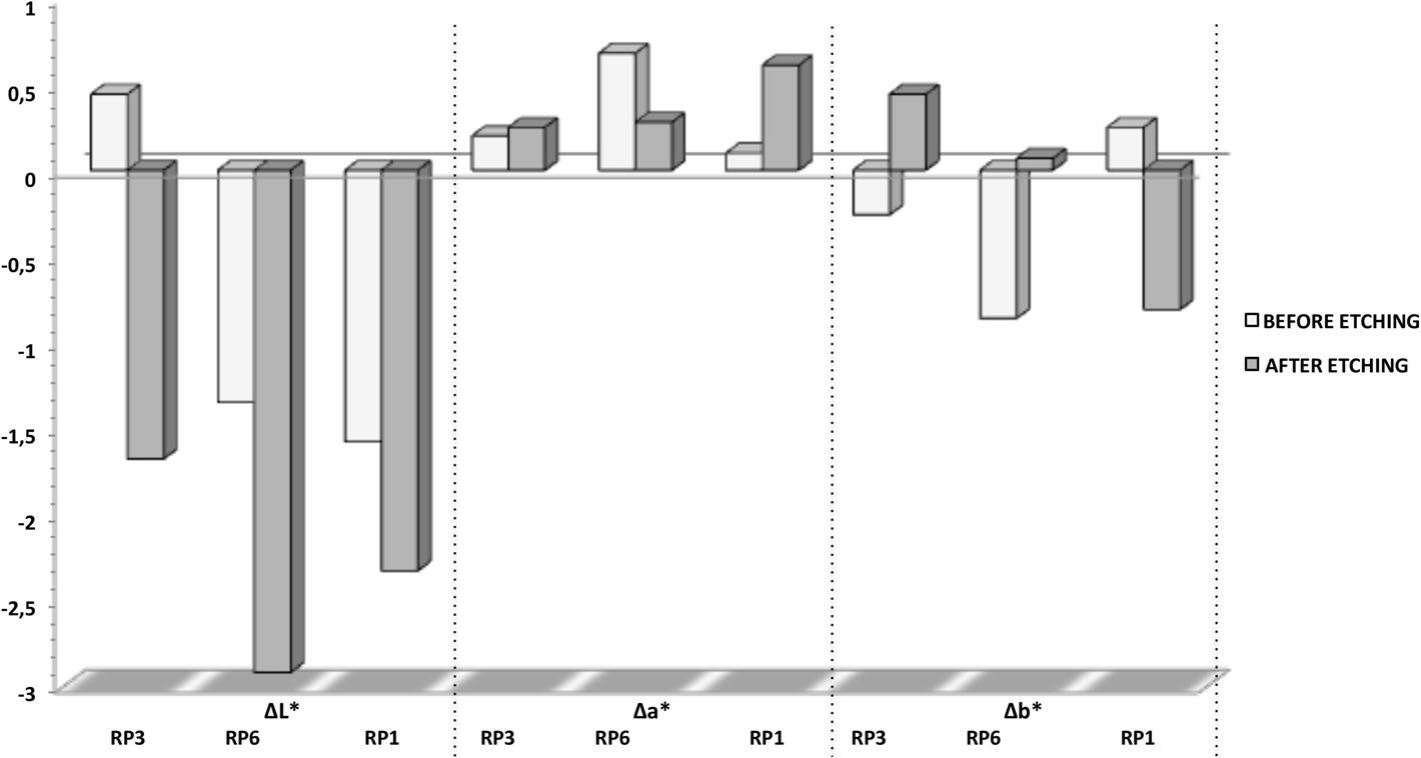
ΔL*, Δa* e Δb* values per experimental group. F test (ANOVA) has not shown a significant difference in the mean values of Δa* (*P* = 0.371) or Δb* (*P* = 0.191). Comparing different propolis nanosuspensions, ΔL* values are significantly different between RP3 before etching and RP3 after etching (*P* = 0.023). There was also a significant difference between RP3 and RP1 (*P* = 0.031) before etching. There was no statistically significant difference for all other comparisons (*P* > 0.05). RP1, 1% of red propolis micellar nanocomposite; RP6, 0.6% of red propolis micellar nanocomposite; RP3, 0.3% of red propolis micellar nanocomposite

Fig. 2 presents the ΔL*, Δa*, and Δb* values according to the processing technique. Except for the group treated with 0.3% micellar nanocomposites loaded with EARP before dentin etching, there was a significant loss of brightness in all groups tested, with greater intensity in the group receiving 0.6% micellar nanocomposites loaded with EARP after dentin etching. There was no significant difference in brightness in all groups treated with propolis after dentin acid etching.

Δa* values indicate variations in the red-green axis, with dominance of the reddish color. In the teeth etched before treatment with micellar nanocomposites loaded with EARP, the values corresponding to the yellow-blue axis (Δb*) moved in the direction of the blue axis with a lower to a higher concentration of micellar nanocomposites loaded with EARP (RP1). When propolis was applied before etching, the RP3 and RP6 groups exhibited a more blueish color and the RP1 group showed a more yellowish color. However, Δa* and Δb* values did not differ significantly between the groups (*P* > 0.05).

**Fig. 3:**
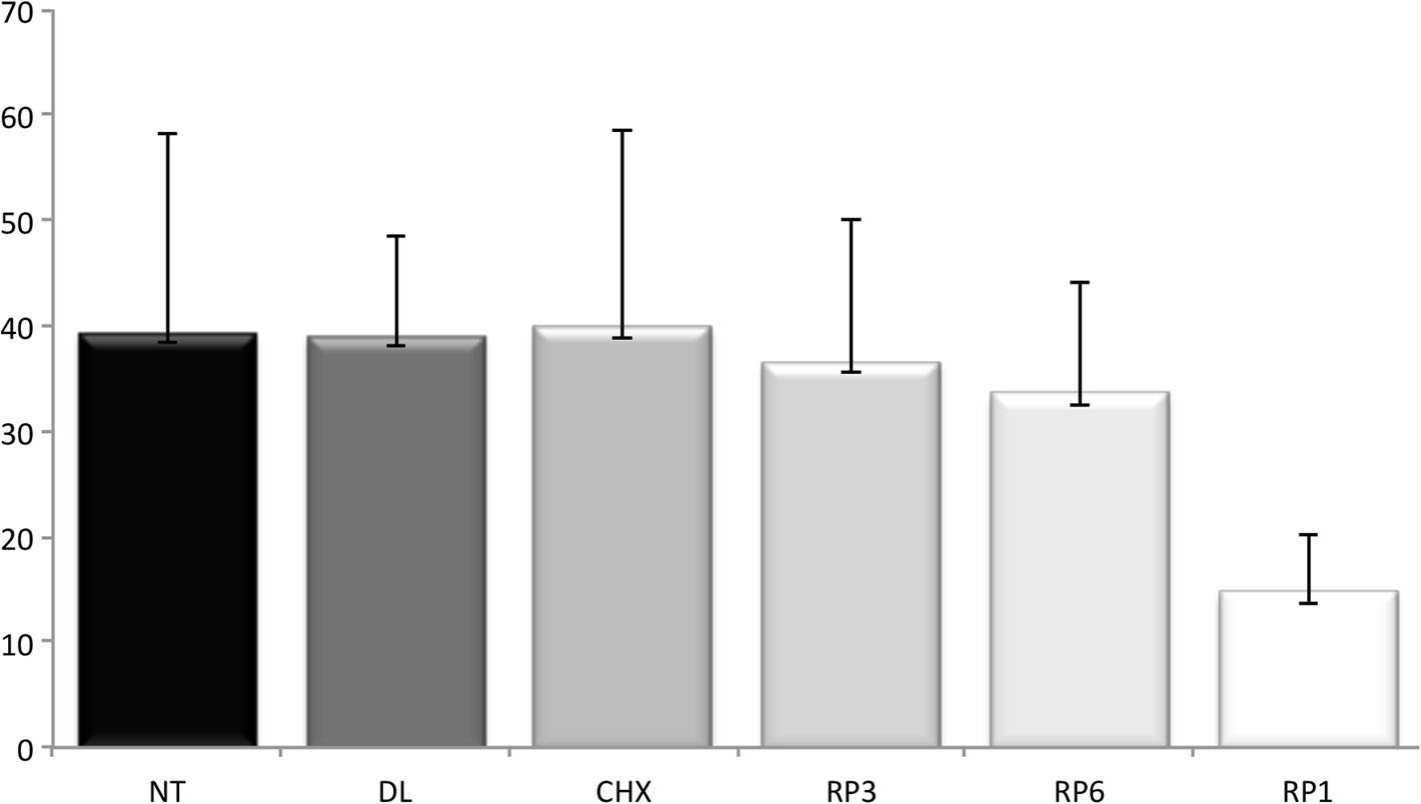
Microtensile dentin bond strength values in MPa. Kruskal-Wallis test for pairwise comparisons of Brazilian red propolis nanosuspensions showed significantly lower microtensile dentin bond strength values for RP1 (*P* = 0.001). RP1, 1% of red propolis micellar nanocomposite; RP6, 0.6% of red propolis micellar nanocomposite; RP3, 0.3% of red propolis micellar nanocomposite; CHX, 2% digluconate chlorhexidine; DL, placebo (micellar nanocomposites without ethyl acetate extract from Brazilian red propolis); NT, no treatment (negative control)

Data from the bond strength tests are shown in Fig. 3. The RP1 group showed a significant reduction in the interface bond strength compared with the other groups (*P* = 0.001). The mean values for the bond strength for the RP3 and RP6 groups were higher than for the RP1 group and similar to the control group, the DL group, and the CHX group. At 0.3 and 0.6% concentrations, micellar nanocomposites loaded with EARP did not induce any decrease in bond strength. However, micellar nanocomposites loaded with EARP at 1.0% concentration reduced the bond strength by 65%.

**Fig. 4:**
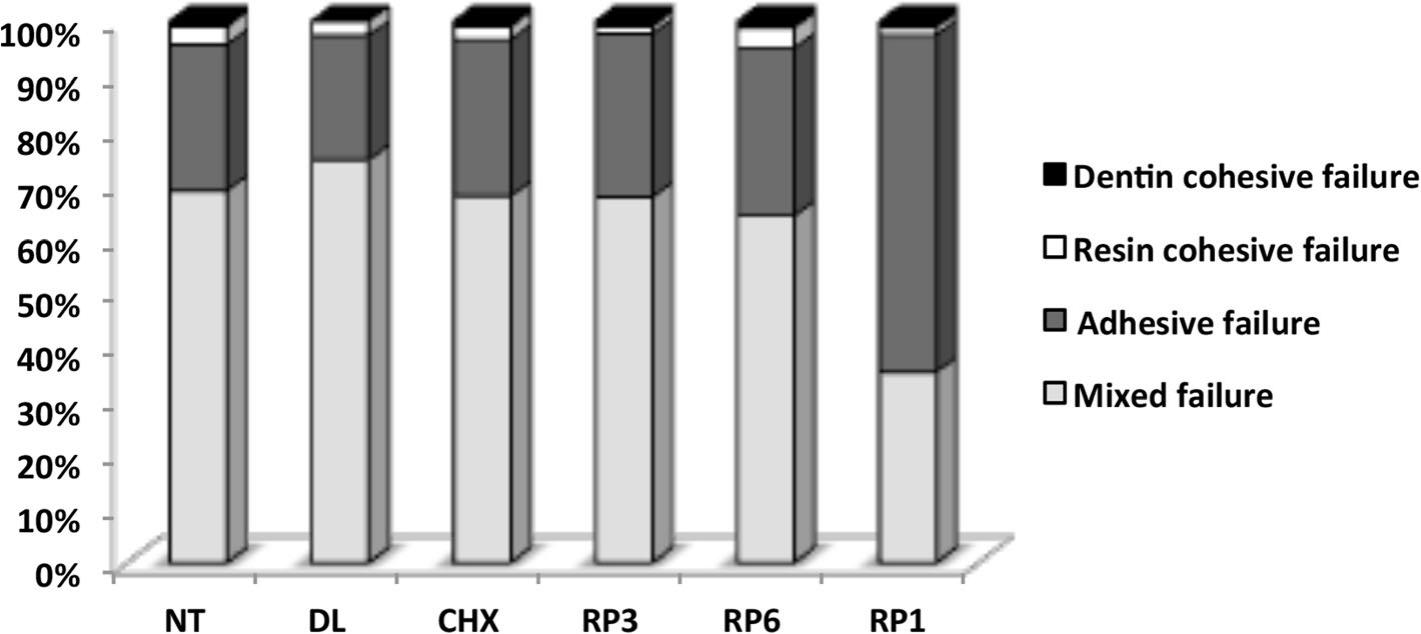
Percentage of the type of fractures per group. The graph shows the predominance of mixed failures. RP1, 1% red propolis nanosuspension; RP6, 0.6% red propolis nanosuspension; RP3, 0.3% red propolis nanosuspension; CHX, 2% digluconate chlorhexidine; DL, placebo (micellar nanocomposites without ethyl acetate extract from Brazilian red propolis); NT, no treatment (negative control)

The fracture mode distribution according to the experimental groups is shown in Fig. 4. The predominant fracture modes were mixed failure, followed by adhesive failure (cohesive failure in the adhesive and/or in the hybrid layer) regardless of the use of propolis micellar nanocomposite or the processing technique. Few cohesive failures in resin and dentin were recorded for the micellar nanocomposites loaded with EARP tested.

**Fig. 5:**
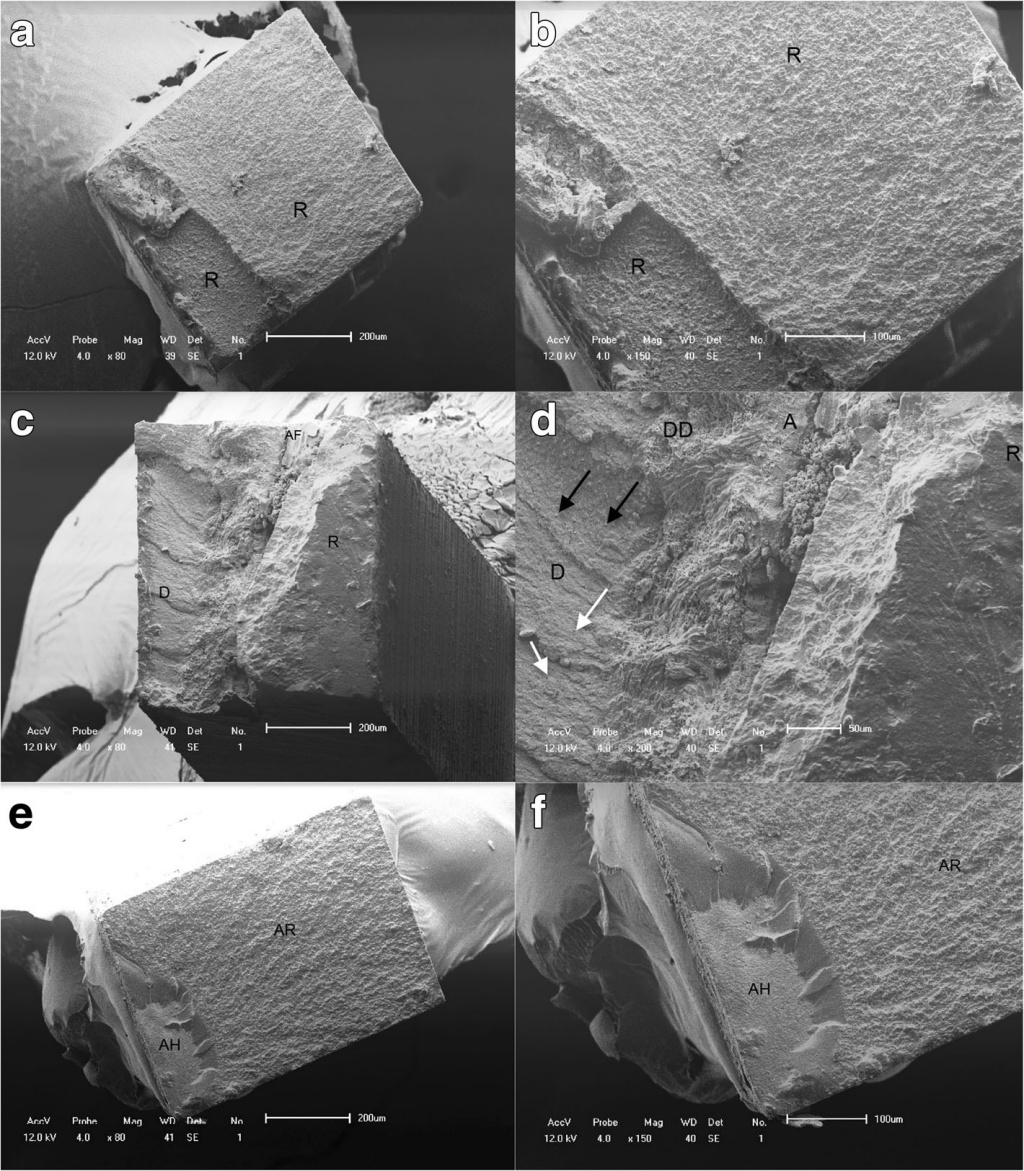
Representative scanning electron micrographs of the fractured specimens. **a** and **b** Scanning electron photomicrographs of the cohesive failure of resin composite. **c** and **d** Mixed fractures. **c** Part of the fracture occurred inside the adhesive layer, probably just under the hybrid layer, because parts of the dentinal tubules are exposed (D-adhesive fracture) and parts are covered by the adhesive layer (A-cohesive fracture) with resin on top of a small adhesive area (R). **d** A mixed fracture shows deep dentin (DD), a thin adhesive film (AF) on dentin, and the presence of opened dentin tubules (white arrows) and fractured resin tags still attached (black arrows). **e** and **f** Adhesive failure: cohesive failure in the bonding resin and/or in the hybrid layer. **e** The fracture that occurred both between the adhesive layer and resin (AR) and under the hybrid layer (AH)

SEM analysis of the dentin and resin surfaces obtained after the bond strength tests revealed the occurrence of mixed fractures (adhesive and cohesive) in almost all the specimens, corroborating the data from the bond strength test. When these specimens were observed under higher magnification, distinct areas of adhesive fractures and cohesive fractures could be observed within the same specimen (Fig. 5).

**Fig. 6:**
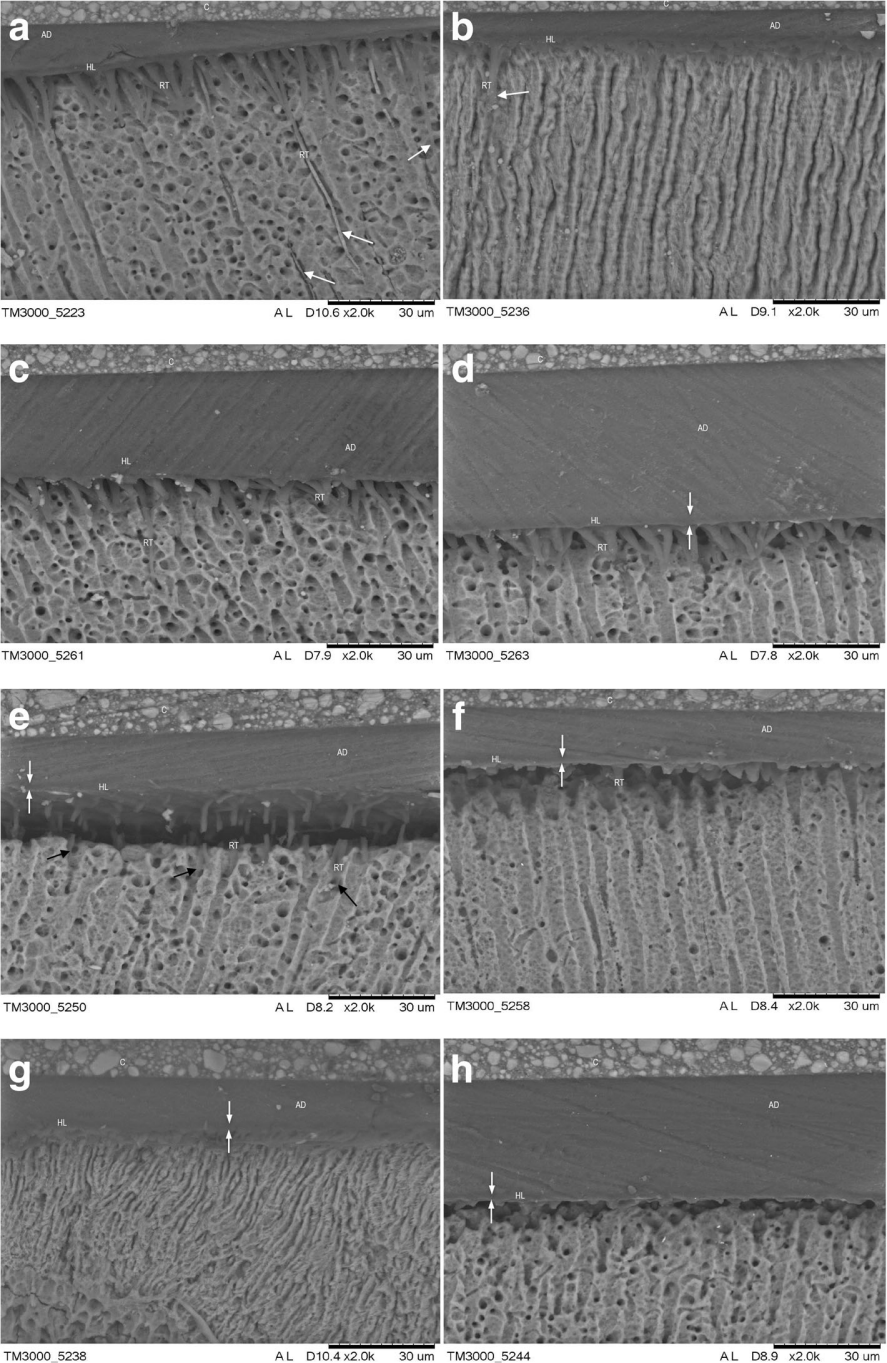
Scanning electron micrographs show the bonded interface of the experimental and control groups. Resin tag quantity and quality match the bond strength results (MPa). White arrows point toward the hybrid layer. C, composite resin; HL, hybrid layer; RT, resin tags; AD, adhesive resin. A uniform hybrid layer with long well-formed resin tags was observed in the control (**a**) and CHX (**b**) groups. RP3 specimens before etching (**c**) and after etching (**d**) show a thin hybrid layer, and relatively shorter resin tags were identified compared with the control group. Representative SEM images of the resin-dentin interface from the RP6 group applied before etching (**e**) and after etching (**f**) show that a very thin hybrid layer was created (white arrows). Short resin tags were identified inside the dentin tubules (E, black arrows). Hardly any hybrid layer could be observed in RP1 specimens. Lack of well-formed resin tags was observed regardless of whether dentin treatment with micellar nanocomposites loaded with EARP was done before (**g**) or after etching (**h**). Only a few very short resin tags can be seen

Fig. 6 shows the micromorphologic aspects of the hybrid layer. The NT and CHX groups show a characteristic image of hybridization of demineralized dentin by adhesive resin with long resin tags deep in the dentinal tubules with lateral branches, which result from the penetration of monomers into the lateral channels that communicate with another dentinal tubule.

In the groups receiving micellar nanocomposites loaded with EARP, the penetration of resin monomers into the dentinal tubules followed by the formation of a zone of interdiffusion dentin/resin (hybrid layer) seems to have been influenced by both the concentration of micellar nanocomposites loaded with EARP and the processing technique (before or after dentin etching). When the propolis micellar nanocomposites were used before dentin etching, application of 0.3% micellar nanocomposites loaded with EARP did not affect the formation of the hybrid layer, similar to the NT and CHX groups. When the concentration of micellar nanocomposites loaded with EARP increased to 0.6 and 1.0%, there was a reduction in the size of the resin tags. When micellar nanocomposites loaded with EARP were applied after dentin etching, it seems that there was substance deposition at the entrance and in the dentin tubules, preventing diffusion of adhesive into the tubules and, consequently, shorter tags or tapered structures formed, restricting the entrance to the dentinal tubules (RP1).

## Discussion

The flavonoids and isoflavonoids (liquiritigenin, pinobanksin, pinocembrin, isoliquiritigenin, daidzein, formononetin and biochanin A) that were identified by the UPLC-DAD assay in both the ethyl acetate extract and micellar nanocomposites loaded with EARP used in this study, combined with other compounds (quercetin, vestitol, and neo-vestitol) present in the ethyl acetate extract may be responsible for the biological activity against the oral pathogens tested [38]. The antimicrobial activity of propolis is usually credited to the phenolic compounds and diterpenic acids in its composition, such as vestitol, neovestitol, quercetin, pinocembrin, galangin, myricetin, kaempherol, apigenin, caffeic acid, caffeic acid phenethyl ester, cinnamic acids, diterpenic acids, and prenylated benzophenones, which also present antibacterial, anti-plaque, and cariogenic activity against microorganisms involved in oral diseases [38–41].

Our results showed that *Lactobacillus acidophilus* ATCC 4356 strain exhibited higher sensitivity to the MNRP than to EARP, with a very low MIC value (< 15.62 μg/mL), probably due to the effect of charge at its surface. Micellar nanocomposites attach more easily to the surface of bacteria because of their positive surface charge, which facilitates the interaction between micellar nanocomposites and the negatively charged external membrane of gram-positive bacteria.

Although acid-producing microorganisms have been isolated from oral microbiota, *Streptococci* and *Lactobacilli* have been considered to be the most significant bacteria involved in the initiation and progression of dental caries, respectively. Several bacteria can be isolated from deep carious lesions; however, *Lactobacilli* were the most commonly isolated microorganisms on dentin from the floor of a cavity preparation [42].

The high sensitivity of *Lactobacillus acidophilus* to MNRP is not surprising, given that this species grows at low pH (4.0), which is the average pH in deep carious lesions. But the MNRP used in this study could lead to a slight increase in the pH of the environment, disturbing the metabolism of *Lactobacillus acidophilus* and contributing to inhibition of the growth of this species*.* Nascimento et al. [31] showed that the pH of suspensions of nanoparticles loaded with Brazilian red propolis was weakly acidic (pH 6.00), which can be attributed to PCL-pluronic copolymers.

Independently of growth medium selectivity for *Streptococcus mutans* and *Candida albicans*, EARP performed better than MNRP, which indicates greater adaptation of these microorganisms to the change in pH of the environment, especially from weakly acid to neutral medium. Although higher MIC values have been recorded for *S. mutans* and *Candida albicans* than for *Lactobacillus* strain, they were three times less than the lowest concentration used (3000 μg/mL). Therefore, our MNRP showed a promising effect for the development of dental materials with antimicrobial activity for cleaning cavities.

The antimicrobial activity of *Streptococcus mutans* found in this study was satisfactory and similar to the results of Righi et al.[16] who reported MIC values for *Streptococcus pyogenes* lower than 256 μg/mL using a methanol extract of red propolis. Oldoni et al. [43] found activity against *S. mutans* using vestitol as a chemical marker with MIC and minimum bactericidal concentration (MBC) values of 31–62 μg/mL and 125–250 μg/mL, respectively. Bueno-Silva et al. [18] demonstrated antimicrobial activity against *S. mutans* with MIC values between 25 and 50 μg/mL for neovestitol and between 50 and 100 μg/mL for vestitol. Purified vestitol and neovestitol presented better MIC and MBC values than the crude extract, chloroform extract, or hexane extracts [18, 43].

Ghasempour et al. [44] isolated *Candida albicans* from dental plaque and caries lesions. The authors suggested that *C. albicans* could lead to caries. The contribution of *C. albicans* to total microbial acid formation appears to be relevant for caries progression; this yeast produces 5-fold more acid per colony-forming unit than lactobacilli at pH 7.0 [45]. *Candida albicans* possesses the capacity to dissolve hydroxyapatite approximately 20-fold when compared with *S. mutans* [46]. Moreover, *C. albicans* has high collagenolytic activity and can adhere to both intact or denaturated exposed dentin collagen [47].

A longitudinal study in children evaluated the antimicrobial efficacy of a dental varnish with 2.5% Brazilian red propolis against *Streptococcus mutans* compared with chlorhexidine 1% and fluoride 5%. Propolis varnish showed consistent reduction in *S. mutans* for up to 6 months in high-risk caries-free children. At day 180, propolis produced significantly lower *S. mutans* levels than fluoride and chlorhexidine [48]. According to research by Bueno-Silva et al. [38], using in vivo animal model studies, flavonoids such as vestitol and neovestitol can inhibit the incidence and severity of dental caries by a downregulation mechanism. Vestitol and neovestitol inhibit the expression of the virulence factor by inhibiting the activity of the enzymes glucosyltransferases D and B, reducing the accumulation of biofilm on the surface of the tooth, and avoiding the formation of caries and dental demineralization. A similar mechanism of caries inhibition has also been demonstrated by Koo et al. [49] using apigenin as an anti-biofilm agent thus avoiding the formation of caries. In this work, apigenin, flavonol, and flavones were active mainly on defective production of the enzymes glucosyltransferases B and C, affecting the pathogenic potential of dental plaque [49]. A potent inhibitory effect on the activity of streptococcal gucosyltransferase enzymes (glucosyltransferases B, C, and D) was compared in an ethanolic extract of Minas Gerais-Brazil propolis and an ethanolic extract from Rio Grande do Sul-Brazil. However, the inhibitory effect on the glucosyltransferase enzymes (B, C, and D) depends on the geographic origin of the propolis [50].

Propolis can be a promising substance for cavity cleaning due to its anti-inflammatory activity [51] and mainly because it is effective against the most of bacteria involved with dental caries process (7,43,48). However, studies have shown different results, which may have been a result of the chemical variability of propolis, the concentration of propolis in the extract, and the solvent used for extraction. Furthermore, the inconsistent results may have been caused by variations in the susceptibility test, culture media, and the time of incubation. The findings of these studies and the present study support the idea that the antibacterial activity depends not only on the origin of the propolis but also on the extract and solvent used [2, 49, 52–55].

Cavity disinfectants are applied before placing the restorative material to reduce or eliminate bacteria from dentin that remains after tooth preparation. Chlorhexidine is a cationic solution with a wide range of antimicrobial activity and is widely used as a cavity disinfectant [56]. The bactericidal effect of the drug is due to the cationic molecule binding to extra-bacterial complexes and negatively charged bacterial cell walls, thereby changing the osmotic balance of the cells. At low concentrations, low molecular weight substances leak out, specifically potassium and phosphorus, with a subsequent bacteriostatic effect. At higher concentrations, CHX has a bactericidal effect as a result of precipitation and/or coagulation of the cytoplasm of bacterial cells followed by cell death [57].

In the present study, we used a 2% CHX solution, which is the gold standard for cavity disinfection in dentistry, because of its long-lasting bactericidal effect on dentin; 0.12% CHX was used for the antimicrobial tests. However, Bidar et al. [58] demonstrated that CHX was effective against *Streptococcus mutans* and *Candida albicans* without significant differences among the different concentrations of CHX used (2, 0.2, and 0.12%).

The partial removal of carious dentin from deep caries lesions leads to significantly improved preservation of pulpal vitality at 5-year follow-up in adults, irrespective of age, supporting the approach of avoiding pulp exposure. This study did not include evaluation of dentin remineralization, but the results of studies [59] suggest that cavity sealing contributes to the remineralization process in inner carious dentin.

The dentin-pulp complex is an important factor in dentin tissue repair. The response of odontoblasts to the inflammatory reaction induced by a carious lesion is the production of tertiary dentin. Partial removal of caries from deep caries lesions followed by cavity sealing promotes a substantial reduction in bacteria, reorganization of the dentin collagen network, narrowing of the dentinal tubules, changes in the tactile consistency (hardness) of dentin and in its mineral content with a significantly higher percentage of calcium and phosphorus and evidence of apatite mineralization [59].

Remineralization of the affected dentin can occur even without any remineralizing material. This strongly suggests that the most important factor is not the material, but the sealing of the cavity, thereby reducing or eliminating the supply of substrate for any remaining microorganisms. We performed dentin pretreatment with micellar nanocomposites loaded with EARP followed by an adhesive restorative procedure. Dentin bonding agents do not induce dentin repair. Although microleakage in composite restorations after antimicrobial treatments can occur [60], some studies showed success after partial removal of carious dentin and cavity sealing with adhesive restorations [25, 58], even after partial caries removal from the pulpal wall followed by adhesive restoration in a single session [61, 62].

This study used the CIE L*a*b * method, which allows numerical color analysis of an object. The CIE L*a*b* system, determined by the Commission International of L’eclaire (CIE) [35] through the CIELAB color space, defines color based on three coordinates: L*, a*, and b*. The L* coordinate (brightness) refers to the level from light to dark, i.e., this coordinate extends from black (L = 0) to white (L = 100); a* and b* values represent the two color axes. The a* coordinate refers to a scale from green to red and varies from − 90 to + 70, with negative values approaching green and positive values for reddish colors; the b* coordinate varies from − 80 to + 100 from blue to yellow with negative values indicating a shift to blue and positive values indicating a shift to yellow. By comparing the initial color reading with the final color reading, it is possible to get the value of Δ*E*, which quantifies the total change in color, but does not qualify it, because it is not possible to indicate on which axis (brightness, red-green, or yellow-blue) the color changes have occurred [36].

To achieve an acceptable aesthetic, the color of dental restorations should match the appearance of the corresponding tissues. The results of this study indicate that the use of micellar nanocomposites loaded with EARP at concentrations of 0.3, 0.6, and 1% did not change the color at the dentin/resin interface in a way that was clinically unacceptable, in agreement with others studies that show clinically acceptable values for color changes in dental restorations [63, 64].

The human eye perceives brightness (L*) more clearly as a result of the higher amount of rod-type cells (responsible for black and white sight) than cone cells (responsible for colored sight) [65]. The color difference between two objects, represented by the Δ*E* value, is considered to be acceptable or not acceptable. However, the Δ*E* values corresponding to this classification are still controversial. Paravina et al. [63] found that when Δ*E* is higher than 3.7, the color difference is easily visible. A value between 3.7 and 1.0 is clinically acceptable and values smaller than 1.0 are not clinically visible. But the study by Douglas et al. [64] reported different data from a survey of dental practice; these authors found that clinicians perceived color changes if Δ*E* ≥ 2.6, but replacement of a restoration because of color changes was only indicated for Δ*E* values higher than 5.5.

The bond strength tests did not show a significant difference between the positive control (CHX) and the negative control (NT). These data are consistent with other studies that have reported the absence of damage to the bond strength of the dentin/resin interface after application of 2% CHX as a cavity disinfectant [66, 67]. Carrilho et al. [68] showed that significant amounts of CHX were retained in dentin independently of the dose of CHX applied (0.2, 2%) or the time (from 30 min to 8 weeks). The substantivity of CHX to dentin probably has an important role in the inhibition of collagen proteases in dentin and in the stability of CHX-treated resin/dentin interfaces [68, 69].

The RP3 and RP6 groups showed microtensile bond strength statistically similar to the control groups and the mean values in MPa were close to those found by Ayar [70] in restorations using the same adhesive system and the same composite resin.

On the other hand, micellar nanocomposites loaded with EARP at a concentration of 1.0% had an adverse effect on the adhesive process, invalidating its use in adhesive restorations. The hybrid layer is a complex composite structure with morphology and properties that are highly sensitive to both the demineralization process and the specific characteristics of the bonding adhesive system [71]. Because of the minimal thickness of the hybrid layer in the RP1 group, it was barely visible, and the shape of the resin tags suggested possible failure in the infiltration of demineralized dentin by the adhesive resin. That was not expected because nanosized drug delivery systems are suitable for improving the bioavailability of poorly water-soluble drugs [23], and in this study, micellar nanocomposites loaded with EARP at concentration of 1% with a particles size of ~ 70 nm in acetone were used, which could flow easily into the dentin tubules. Chen et al. [9] showed that applying 10% propolis ethanolic extract to dentin leads to deposition of resins and waxes from propolis on the dentin surface and into the dentinal tubules. It can block the entrance to the dentin tubules and cover the dentin surface, interfering with the proper formation of the hybrid layer and resin tags. Although the concentration used in this study was 10 times lower than that used by Chen et al. [9], dentin hybridization was compromised followed by reduced dentin-resin bonding.

Polymeric micelles serve as nanoscopic drug carriers [72] and consist of a core and shell structure; the inner core is the hydrophobic part of the block copolymer, which encapsulates the poorly water-soluble drug, whereas the outer shell or corona of the hydrophilic block of the copolymer protects the drug from the aqueous environment, keeping them stable. The core can sometimes be made up of a water-soluble polymer that is rendered hydrophobic by the chemical conjugation of the water-insoluble drug [73, 74].

For this study, polymeric micelles were prepared from PCL and Pluronic F108 copolymer, biocompatible and biodegradable block copolymers that may remain intact for long duration under sink conditions and may also slowly release drugs. The failure noticed in the RP1 group may be associated with the higher concentration of micellar nanocomposite and the consequent increase in the viscosity.

Donor-acceptor interactions between a solid surface and an organic liquid lead to the creation of surface charge and counter ions in the liquid [75]. Zeta potential, a scientific term for the electrokinetic potential in colloidal systems [76] and nano-medicines, and the particle size exert a major effect on the various properties of nano-drug delivery systems [77] and can be an indicator of stability. High electric charge (+ 30 mV), positive or negative, on the surface of the nanoparticles prevents aggregation of the nanoparticles because of the strong repellent forces among the particles [78, 79]. The highest concentration of micellar nanocomposites loaded with EARP (RP1 group) may have been responsible for a reduction in the zeta potential of the micellar PCL/Pluronic nanocomposite and led to the formation of nanoclusters, which would increase the viscosity and act as physical blockers contributing to less penetration of the adhesive system into the dentin tubules.

## Conclusion

Micellar nanocomposites loaded with EARP at concentrations of 0.3 and 0.6% applied as a cavity cleanser do not compromise the aesthetics or microtensile bond strength of the dentin/resin interface. The EARP is rich in flavonoids and isoflavonoids and both EARP and MNRP showed antimicrobial activity for the main agents causing dental caries (*Streptococcus mutans* and *Lactobacillus acidophilus)* and for *Candida albicans*.

## Abbreviations

ANOVA: Analysis of variance
CHX: Chlorhexidine gluconate
EARP: Ethyl acetate extract of Brazilian red propolis
MIC: Minimal inhibitory concentration
MNRP: Micellar nanocomposites loaded with EARP
NT: No treatment
PCL: Poly-ε-caprolactone
SEM: Scanning electron microscopy
UPLC-DAD: Ultra-performance liquid chromatography coupled with a diode array detector
μTBS: Microtensile bond strength
